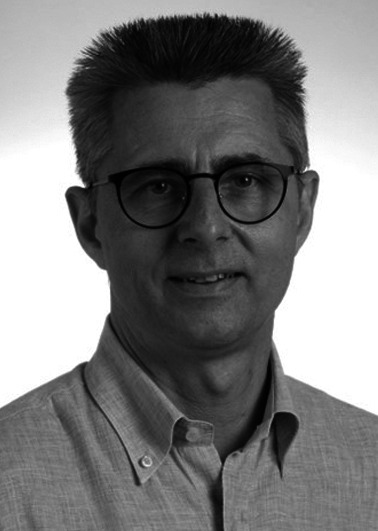# The Nickel Age in Synthetic Dual Photocatalysis: A Bright Trip Toward Materials Science

**DOI:** 10.1002/cssc.202201094

**Published:** 2022-08-04

**Authors:** Miriam Marchi, Giuseppe Gentile, Cristian Rosso, Michele Melchionna, Paolo Fornasiero, Giacomo Filippini, Maurizio Prato

**Affiliations:** ^1^ Department of Chemical and Pharmaceutical Sciences CENMAT Center of Excellence for Nanostructured Materials INSTM UdR Trieste University of Trieste Via Licio Giorgieri 1 34127 Trieste Italy; ^2^ Consorzio Interuniversitario Nazionale per la Scienza e Tecnologia dei Materiali (INSTM) Unit of Trieste via L. Giorgieri 1 34127 Trieste Italy; ^3^ Center for Cooperative Research in Biomaterials (CIC biomaGUNE) Basque Research and Technology Alliance (BRTA) Paseo Miramón 194 20014 Donostia San Sebastián Spain; ^4^ Basque Fdn Sci, Ikerbasque 48013 Bilbao Spain

**Keywords:** dual catalysis, nickel, photochemistry, photoredox catalysis, synthetic methods

## Abstract

Recently, the field of dual photocatalysis has grown rapidly, to become one of the most powerful tools for the functionalization of organic molecules under mild conditions. In particular, the merging of Earth‐abundant nickel‐based catalytic systems with visible‐light‐activated photoredox catalysts has allowed the development of a number of unique green synthetic approaches. This goes in the direction of ensuring an effective and sustainable chemical production, while safeguarding human health and environment. Importantly, this relatively new branch of catalysis has inspired an interdisciplinary stream of research that spans from inorganic and organic chemistry to materials science, thus establishing itself as one dominant trend in modern organic synthesis. This Review aims at illustrating the milestones on the timeline evolution of the photocatalytic systems used, with a critical analysis toward novel applications based on the use of photoactive two‐dimensional carbon‐based nanostructures. Lastly, forward‐looking opportunities within this intriguing research field are discussed.

## Graphical abstract

**Double trouble**: Dual Ni/photoredox catalysis is a prominent approach to forge valuable C−C and C−heteroatom bonds of synthetic relevance. This Review summarizes the most relevant examples within this field, highlighting the great evolution of the different photocatalytic systems synergistically employed together with the Ni catalyst. These span from metal‐based complexes to organic dyes, along with heterogeneous semiconductors.

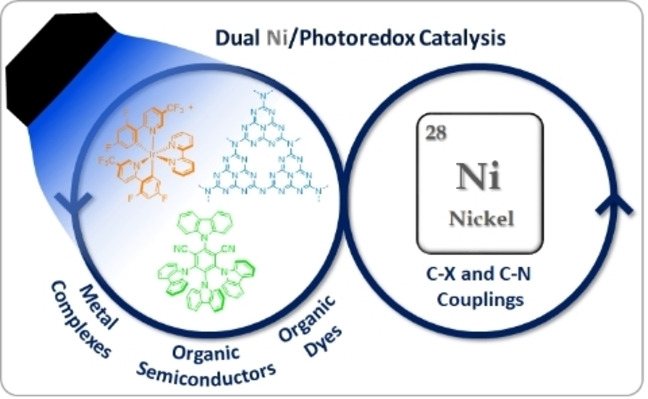

## 1 Introduction

**Figure 1:**
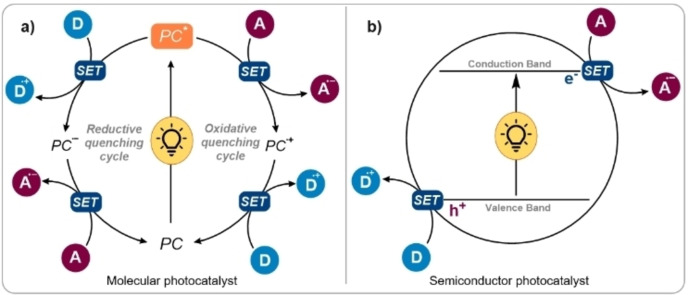
(a) General reductive and oxidative quenching cycles exploited in homogeneous photoredox catalysis. (b) General catalytic mechanism of heterogeneous photoredox catalysis. PC: photoredox catalyst; D: electron donor; A: electron acceptor.

Over the past decades, visible light photocatalysis has gained great attention as a useful tool to aid the development of new reactivities in organic synthesis.^[1]^ Differently from conventional catalysis, photocatalysis exploits the formation of reactive intermediates via photoinduced electron‐, energy‐, or atom‐transfer processes.[^2^, ^3^] A widespread photocatalytic approach is represented by the use of photoredox catalysis, which typically exploits the light‐induced redox abilities of molecular chromophores (namely photoredox catalysts: PCs) to generate reactive radicals under very mild operative conditions.[^4^, ^5^] Specifically, excited‐state photoredox catalysts (PCs*) can act both as strong oxidants and strong reductants towards suitable substrates, thereby providing access to unique reaction environments.^[6]^ From a mechanistic point of view, a PC reaches an electronically excited state upon absorption of light with wavelength equal or higher than the state (PC and PC*) energy difference (Figure 1a). Then, under appropriate conditions, the so‐obtained excited catalyst (PC*) is able to engage in sequential single‐electron transfer (SET) events, hence returning to its ground state either by a reductive or an oxidative quenching cycle. In the former, the PC* promotes a SET oxidation of an electron‐rich donor species (D). Afterwards, the reduced catalyst is restored to its ground state through a second electron transfer process by reducing an electron‐deficient acceptor species (A). The opposite sequence occurs in the oxidative quenching cycle, where the PC* donated an electron to A, and subsequentially the oxidized photocatalyst is reduced by D. This exceptional electronic duality of PCs is instrumental for the development of novel attractive carbon–carbon and carbon–heteroatom bond‐forming transformations.[^3^, ^4^, ^5^]

Recently, the concurrent efforts by MacMillan and co‐workers, Stephenson and co‐workers, and Yoon and co‐workers, along with other contributors, have revived interest in the field of metal‐based photoredox catalysis within the synthetic community.[^4^, ^7^, ^8^, ^9^, ^10^]

Among the reasons for this rapid growth, versatility and effectiveness of photo‐active transition metal complexes in orchestrating a wide range of organic transformations have been of great importance.[^7^, ^11^] The traditional and best‐known transition metal‐based PCs consist of ruthenium and iridium complexes. As an example, [Ru(bpy)_3_]Cl_2_ (bpy=2,2’‐bipyridine), which is a coordinatively saturated ruthenium‐pyridyl complex, has been widely studied and employed as a PC due to its well‐suited photoredox features.[^4^, ^6^] More specifically, its photocatalytic activity can be tuned by varying the electronic nature of the organic ligands.[^12^, ^13^] In fact, when the electron density at the metal atom is enhanced, the oxidative power of the PC decreases whereas its reductive power increases. This aspect is at the basis of the versatility of the PCs, whereby the synthetic design of the metal complex structure (i. e., what metal and what ligands must be chosen) is rationalized on account of the redox potentials of the substrates used (A and D) for the desired chemical transformation. In this regard, it must be highlighted that the synthesis of both the ligands and the final metal complexes often requires tedious multi‐step routes. Moreover, reliance on noble metal‐based complexes has been constantly declined over recent years due to cost, availability and toxicity, therefore discouraged by the modern guidelines towards implementation of sustainable chemical production schemes.[^5^, ^14^] As a new trend of research, cheaper, abundant and less harmful classes of metal‐free PCs are making their way as valuable alternatives for achieving greener chemical productions.[^14^, ^15^] Molecular organic dyes have emerged as a promising class of PCs, mainly considering their availability along with low cost and toxicity. Moreover, the photoredox properties of these purely organic PCs can be easily adjusted through ad hoc functionalization of their molecular structure.[^5^, ^7^, ^16^, ^17^] In the last years, various dyes have been investigated in organic photoredox catalysis, all typically characterized by a highly functionalized aromatic core, namely xanthenes, perylenediimide, phenols, thiazines, acridinium salts, cyanoarenes, flavins, and diaryl ketones, among others.[^5^, ^14^, ^17^, ^18^, ^19^] In some cases, organic dyes are used in relatively high catalytic loading (up to 20 mol %), and they might also be constrained by tedious multi‐step preparations, inability to be recycled, and instability under certain reaction conditions.[^5^, ^14^] An extremely intriguing application is to couple photoredox catalysis with a non‐photochemical second catalytic route to exploit possible synergy towards more challenging chemical conversions or multiple products. Within this strategy, also known as “dual catalysis”,^[20]^ reactive intermediates obtained through a photoredox process may, for example, be involved in organo‐ or transition‐metal catalytic cycles for the preparation of high‐value organic compounds.[^20^, ^21^] Since an early work by Sanford and co‐workers in 2011,^[22]^ followed by independent reports from MacMillan and co‐workers, the application of photoredox catalysis to the field of metal‐mediated processes, defined more simply as metallaphotoredox catalysis, has received considerable interest.[^4^, ^23^, ^24^]

**Figure 2:**
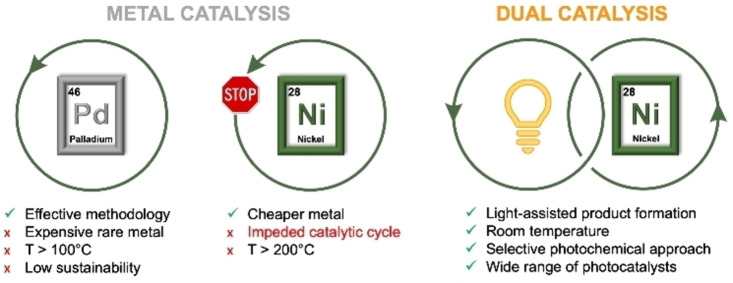
Comparison between metal and dual catalysis to drive C−C and C−X coupling reactions.

One popular example consists in the combination of a visible‐light molecular PC, typically an Ir or Ru polypyridyl complex, with a nickel‐containing catalyst, which has given access to the development of many challenging C−C and C−heteroatom couplings under mild reaction conditions.^[25]^ These transformations include also photo‐driven enantioselective organic reactions, which drive the formation of enantioenriched molecules.[^26^, ^27^, ^28^, ^29^, ^30^, ^31^] The high abundance and low cost of nickel make this metal unique and more attractive than other conventionally used transition metals, such as palladium. The chemistry of nickel also offers a great advantage: in contrast to palladium, where only 0 and +2 oxidation states are usually accessible, nickel‐based catalysis may involve all oxidation states between 0 and +3 when an external PC participates in the process.^[32]^ This dual photochemical approach opens the way to a wide range of synthetic avenues. Remarkably, the presence of the PC is essential as otherwise these routes would not be viable in mild conditions with the sole use of Ni catalysis (Figure 2).

**Figure 3:**
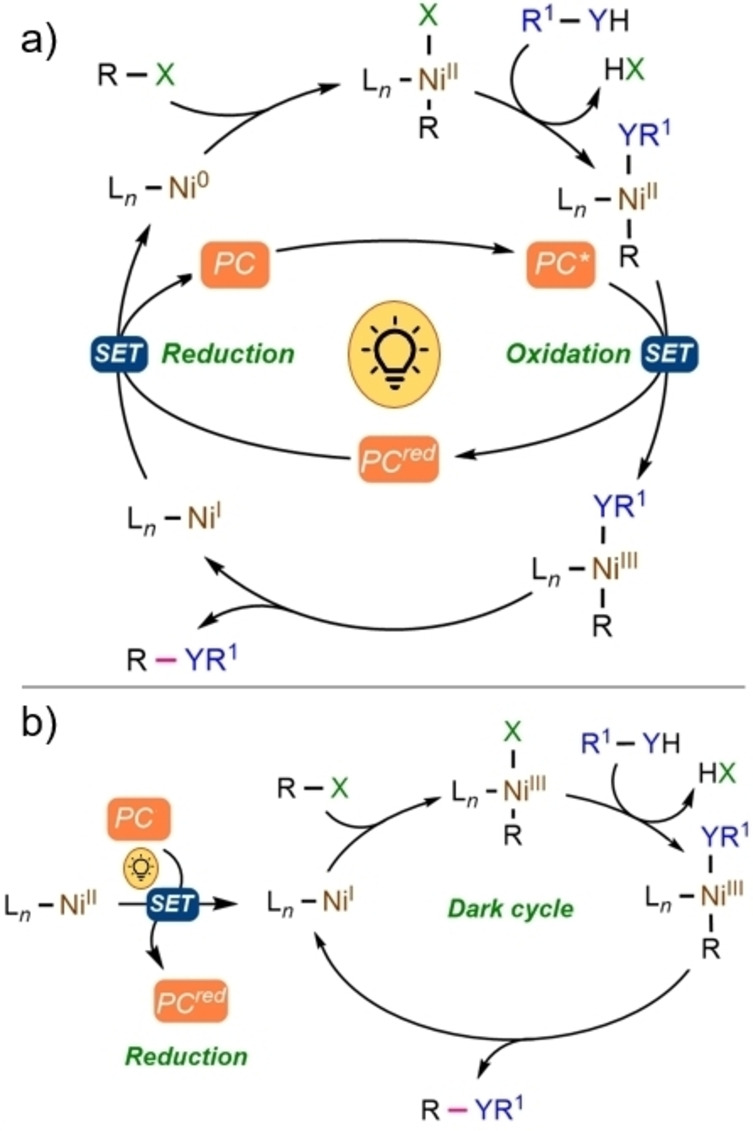
(a) General Ni^0^–Ni^II^–Ni^III^ mechanism. (b) General Ni^II^–Ni^I^–Ni^III^ mechanism.

Mechanistically, the merger of a nickel‐based species with a photoredox catalyst can be schematized as two interconnected catalytic cycles, as depicted in Figure 2, which can evolve according to two main mechanistic scenarios. Within the first proposed mechanism (Figure 3a), a Ni^0^ species is transformed into a Ni^II^ complex through an oxidative addition step. Then, a SET oxidation process of Ni^II^, driven by the excited PC may form a Ni^III^ complex, which undergoes a more facile reductive elimination than a Ni^II^ species. Lastly, the reduction of Ni^I^ species closes the combined catalytic cycle.[^33^, ^34^] In alternative to this route, a second possible scenario, also supported by density functional theory (DFT) calculations, entails the light‐mediated formation of a reactive Ni^I^ species from a Ni^II^‐based pre‐catalyst, which is then capable of initiating a self‐sustaining Ni^I^/Ni^III^ operative cycle (Figure 3b).^[35]^

**Figure 4:**
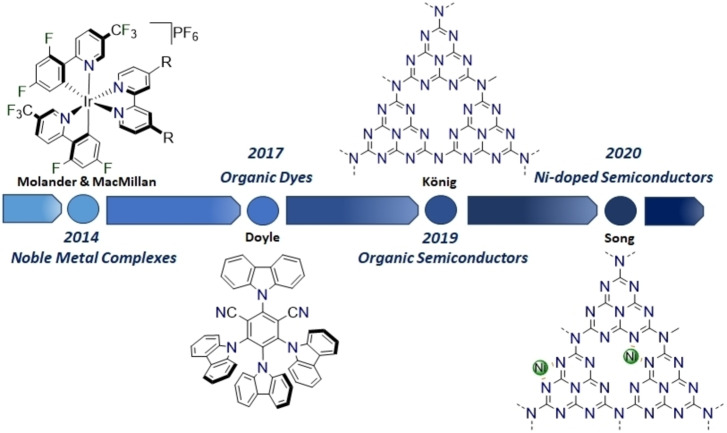
Evolution of the photoredox catalysts: from noble metal complexes to two‐dimensional organic semiconductors.

In this type of process, the presence of the PC ensures the perpetuation of the reactivity through a continuous reduction of the eventually regenerated Ni^II^ catalyst into the active Ni^I^ intermediate.[^36^, ^37^, ^38^, ^39^] Nevertheless, it is important to emphasize that the detailed mechanism is still debated, and a better understanding of this process is required. Importantly, it is likely that the actual mechanism of Ni/photoredox catalysis strictly depends on the nature of the reaction components and conditions.^[40]^ In the early studies on Ni/photoredox dual catalysis, either Ru‐ or Ir‐based complexes were used as PCs (Figure 4).[^36^, ^41^] Successively, these metal‐containing systems have been replaced with more sustainable photocatalytic alternatives, namely organic dyes.^[42]^ However, molecular PCs are not generally ideal candidates for attracting industrial interest because they present limitations both in terms of recovery and recycling due to their homogeneous nature in relation to solution‐phase catalysis.[^5^, ^42^] A strategy to overcome such drawbacks relies on the use of semiconductor‐based heterogeneous photoredox catalysts, which present easy preparation, low cost, excellent optoelectronic features, and recyclability.[^43^, ^44^]

When a semiconductor absorbs photons of sufficient energy, excitation of electrons from the valence band (VB) to the conduction band (CB) occurs with consequent charge separation (electrons and holes). If the potential energies of the VB and the CB are suitable, the as‐formed holes (h^+^) and electrons (e^−^) are thermodynamically competent to respectively oxidize donors (D) and reduce acceptors (A) via SET, provided that the VB and CB energy levels are higher (in absolute value) with respect to the redox potential of D and A (Figure 1b).^[43]^ Over the last years, transition metal oxides and dichalcogenides, semiconductor quantum dots (QDs), metal–organic‐frameworks (MOFs), perovskites, and different types of carbon‐based nanomaterials have been efficiently employed for this synthetic purpose.[^43^, ^45^] Among the numerous heterogeneous semiconductors, graphitic carbon nitride (*g*‐CN) and its derivatives, which are stable nanomaterials capable of absorbing visible‐light, represent one of the most investigated classes of heterogeneous PCs.[^40^, ^45^, ^46^] Apart from the simplicity in the preparation and the typically narrow bandgap, the versatility of the *g*‐CN semiconductor class resides in a rich functionalization potentiality and morphology adjustment that are exploited to drive desired photocatalytic processes.[^47^, ^48^, ^49^, ^50^]

An additional appealing feature derives from the ability of *g*‐CN to act as single‐atom host, whereby single metal ions can be captured and stabilized by means of the extensive availability of binding N atoms. Single‐atom catalysts (SAC) are currently one of the frontiers in heterogeneous catalysis, where the robustness of heterogeneous systems is fruitfully combined with the ideal metal utilization of homogeneous metal catalysts, also allowing catalyst recovery.[^51^, ^52^, ^53^] While SACs are widely used in several catalytic processes, especially related to energy applications, their dual catalysis nature is seldom recognized. Still, from one viewpoint, they can be considered the ideal example of fully heterogeneous dual catalysts, with the PC and the metal complex site all integrated in a “one‐package” catalyst, the former absorbing light to alter the electronic state of the metal site by means of electron donation/abstraction. Lastly, purely nickel‐catalyzed methodologies, which do not need an external photocatalyst, have been also investigated. In this case, these approaches involve a direct photoexcitation of nickel complex under UV light irradiation.[^54^, ^55^, ^56^] Nevertheless, these last synthetic methodologies will not be discussed hereafter. Indeed, this Review aims at providing an instructional and historical overview on the recent findings on the application of dual Ni/photoredox catalysis in organic synthesis. Specifically, the chemical identity of the used PCs has rapidly progressed in recent years, moving from noble metal‐based complexes to photoactive carbon‐based nanostructures (Figure 4). Thus, the purpose is to provide an ensemble of guidelines for this flourishing research branch to inspire and stimulate the development of forthcoming useful methodologies and encourage the adoption of detailed mechanistic investigations during the catalytic application development.

## 2 Dual Ni/Metallaphotoredox Catalysis

**Figure 5:**
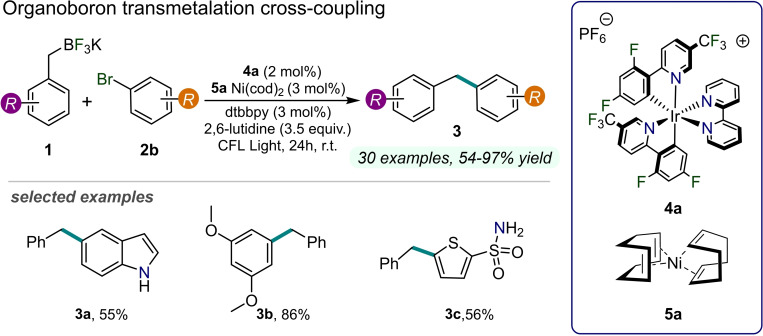
Dual Ni/Ir‐photoredox‐catalyzed C−C coupling between trifluoroborates **1** and aryl bromides **2**, described in Ref. [34]. cod=1,5‐cyclooctadiene; tbbpy=4,4’‐di‐*tert*‐butyl‐2,2’‐dipyridyl; CFL=compact fluorescent lamp.

As previously mentioned, many of the most commonly employed metal‐based visible‐light photoredox catalysts are polypyridyl complexes of ruthenium and iridium. These photoactive species have been exploited in cross‐coupling reactions commonly involving aryl halides (e.g., iodides, bromides and chlorides **2a‐c**), hence affording a variety of coupling products. In this regard, a milestone within this field is represented by the work of Molander and co‐workers published in 2014. The authors reported a procedure allowing the direct exploitation of benzyltrifluoroborate derivatives **1** in C−C cross‐coupling reactions, using the pioneering combination of nickel chemistry and photoredox catalysis (Figure 5).^[34]^ In this study, a Ni^0^ catalyst **5 a**, a bipyridine ligand, 2,6‐lutidine, and an Ir^III^‐based photocatalyst **4 a** are employed to drive the oxidative C(sp^2^)−C(sp^3^) cross‐coupling reactions between **1** and aryl bromides **2 b** to give compounds **3** upon visible light irradiation (up to 99 % yield). Specifically, this protocol circumvents the well‐known slowness of the two‐electron transmetalation approach, leading instead to the conversion of **1** into carbon‐centered radicals thanks to photochemical activity of the Ir^III^‐based PC. Indeed, the hypothesized reaction mechanism starts with the oxidative addition of **2 b** to the Ni^0^ catalyst forming a Ni^II^ species. Concomitantly, a benzylic radical is formed from **1** through photoinduced SET process mediated by the excited **4 a**. This open shell species is then trapped by the Ni^II^ complex, yielding a high‐valent Ni^III^ intermediate. Lastly, Ni^III^ species can undergo reductive elimination to obtain the desired product **3** along with a Ni^I^ species. Remarkably, the authors developed an asymmetric version of this procedure by employing a suitable enantiopure chiral ligand. In this manner, racemic alkylborate reagents can be involved in stereoconvergent transmetalation reactions forming model enantioenriched coupling products.

**Figure 6:**
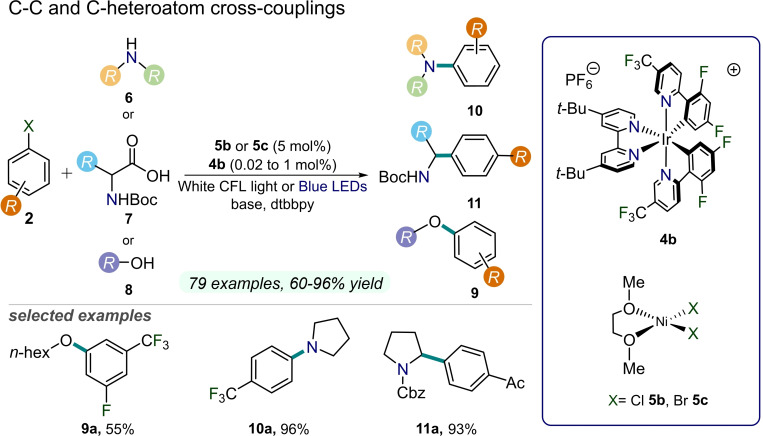
Dual Ni/Ir‐photoredox‐catalyzed C−C and C−heteroatom cross couplings between aryl halides **2** and amines **6**, amino acids **7**, or alcohols **8**, described in Refs. [23, 24, 33]. Dtbbpy=4,4’‐di‐*tert*‐butyl‐2,2’‐dipyridyl; CFL=compact fluorescent lamp. Boc=*tert*‐Butyloxycarbonyl. Cbz=benzyloxycarbonyl.

In the same year, MacMillan and co‐workers described that the synergistic combination of an Ir‐based photocatalyst **4 b** with a nickel catalyst **5 b** allows for the development of a C(sp^2^)−C(sp^3^) decarboxylative arylation reaction between carboxylic acids **7** and aryl halides **2**. This synthetic protocol enables the preparation of relevant protected benzyl amines **11** (e.g., Boc or Cbz) using simple and accessible starting materials (up to 93 % yield, Figure 6).^[23]^ Indeed, carboxylic acids are appealing radical precursors for their widespread availability and synthetic accessibility. In addition, the authors also described that the same catalytic system is capable of driving oxidative C(sp^3^)−H cross‐coupling reactions of dimethylanilines with a variety of aryl halides **2**, thus proving the wide versatility of the developed metallaphotoredox approach.

Alkyl bis(catecholato)silicates were also investigated as radical precursors in Ni/photoredox‐catalyzed C(sp^2^)−C(sp^3^) cross coupling reactions. Interestingly, these substrates possess lower oxidation potentials than **1** and **7**, thus favoring the photochemical radical initiation step.[^36^, ^57^]

A remarkable example targeting the formation of challenging C−O bonds between **2** and alcohols **8** under blue light irradiation was achieved by MacMillan and co‐workers in 2015 (Figure 6).^[24]^ Mechanistically, the oxidative addition of bromoarene **2 b** to a Ni^0^ complex leads to a Ni^II^ aryl complex. Subsequentially, the formation of the transient Ni^III^ aryl alkoxide intermediate occurs by the action of the excited Ir‐based PC **4 b**, which may effectively oxidize the Ni^II^ complex, as confirmed by cyclic voltammetry measurements. The as‐formed Ni^III^ species undergoes reductive elimination to forge the desired aryl ether product **9** and a Ni^I^ complex. Lastly, the reduced PC donates an electron to the Ni^I^ species, thus completing the catalytic cycle. Importantly, a broad array of primary and secondary alcohols **8** have been transformed into the desired products **9** in high overall yields (up to 96 %). This study paved the way for the subsequent development of photocatalytic C−heteroatom coupling reactions, recognizing that photo‐driven access to the elusive Ni^III^ intermediate was the key to complete the mechanistic cycle towards an exothermic reductive elimination step.

The flexibility of Ni/photoredox dual‐catalysis is further demonstrated by the C−P bond formation reactions between diarylphosphine oxide and aryl iodides, which was catalyzed by a Ru‐based PC along with a Ni^0^ derivative.^[58]^ Notably, the authors presented a wide reaction scope, using catalyst under visible‐light irradiation.

In 2016, MacMillan and co‐workers presented a protocol for the direct aryl amination reactions of **2** with simple amines **6** to yield valuable aniline products **10** (up to 96 % yield), employing a nickel/photoredox dual‐catalyzed approach (Figure 6).^[33]^ The authors employed (1,2‐dimethoxyethane)nickel dibromide catalyst (NiBr_2_ ⋅ glyme) **5 c**, an Ir^III^ photocatalyst **4 b**, a base, and a supporting ligand to forge the desired C−N bonds under blue light irradiation. It is worth noting that this approach was successfully employed for the preparation of drug‐like aniline compounds of pharmaceutical interest in a high‐throughput method.

By combining an Ir‐based PC with a Ni catalyst, the highly chemoselective cross‐coupling reaction between aryl amines and aryl halides **2** under blue light irradiation was also attained.[^40^, ^59^] Subsequently, the same research group developed an Ir/Ni‐dual‐catalyzed C−S bond forming protocol to couple (hetero)aryl iodides with thiols, where the C−S cross‐coupling relies on a mechanism involving the SET oxidation step, mediated by the Ir‐based PC, to generate thiyl radicals from thiols.^[60]^

**Figure 7:**
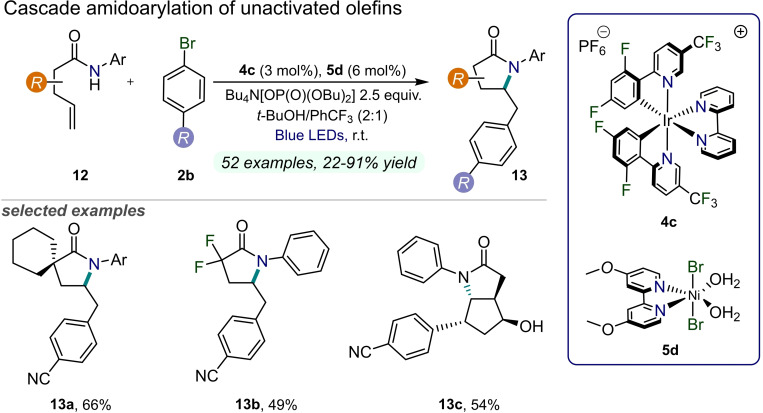
Dual Ni/Ir‐photoredox‐catalyzed amidoarylation of unactivated olefins **12**, described in Ref. [61].

In 2019, Molander and co‐workers described a highly diastereoselective arylation of amides bearing a terminal olefin moiety **12** by concerted photoredox proton coupled electron transfer (PCET) with nickel catalysis to forge useful highly functionalized five‐membered heterocyclic systems **13** (Figure 7).^[61]^ This process has been carried out under blue LED irradiation employing a Ni(dMeObpy)(H_2_O)_2_Br_2_ catalyst **5 d** and an Ir photocatalyst **4 c** in relatively high loading. Mechanistic studies revealed that the initial formation of an amidyl radical conducted via PCET is followed by a rapid 5‐*exo*‐trig cyclization. Then, the alkyl radical enters the nickel‐catalytic cycle and the Ni^I^ species undergoes oxidative addition with aryl halide to Ni^III^ intermediate, which is subjected to reductive elimination. Substrate scope has been expanded not only towards amides, but also carbamates and ureas could be converted with the studied methodology into pharmaceutically relevant products.

These examples clearly indicate that, while in Pd chemistry the C−C/C−heteroatom bond formation is triggered by a careful tuning of the ligands structure, the Ni‐based dual photocatalytic strategy is based on the advantageous destabilization of a simpler Ni‐amido species by means of electron‐transfer through the photoactive Ir complex.

## 3 Dual Ni/Organophotoredox Catalysis

As mentioned above, organic dye‐based PCs represent a better suited choice from a cost perspective. Organic dyes are highly conjugated organic molecules with strong absorption in the visible region and prominent redox character and include compounds such as 9‐mesityl‐10‐methylacridinium (9‐PhAcr), Eosin Y, or 1,2,3,5‐tetrakis(carbozol‐9‐yl)‐4.6‐dicyanobenzene (4CzIPN). They present high stability in solution and excited‐state redox potentials comparable to those of metal‐based PCs, hence making them attractive in photoredox catalysis.^[62]^ Organic dyes have typically an easy preparation, and their spectroscopical and electronic properties are extremely tunable and versatile through synthetic modifications.[^5^, ^14^] The use of organic chromophores in photoredox catalysis for synthetic chemistry is recent and still not completely developed. Only in recent years, organic photocatalysts have been exploited for generating carbon‐ and heteroatom‐centered radicals and charged open shell species in organic photocatalysis.^[12]^ In this section, the main examples of combination of organophotoredox catalysis with nickel catalysis will be described.

**Figure 8:**
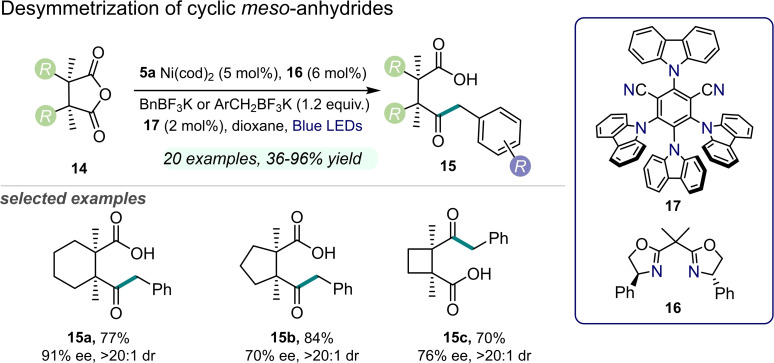
Dual Ni/organophotoredox‐catalyzed desymmetrization of *meso*‐anhydrides **14**, described in Ref. [63]. cod=1,5‐cyclooctadiene.

Doyle and co‐workers reported in 2017 the enantioselective desymmetrization of cyclic *meso*‐anhydrides **14** with benzyl trifluoroborates **1** by one of the very first examples of dual nickel/organophotoredox catalysis (Figure 8).^[63]^ The authors employed a merged catalytic manifold constituted by a Ni^0^ complex **5 a**, a chiral bisoxazoline ligand **16**, and a visible light‐absorbing dye, namely 4‐CzIPN (**17**). In this way, the starting achiral anhydrides **14** were converted into the corresponding enantioenriched keto‐acids **15** under blue light irradiation in good to high yield and selectivity [up to 90 % yield, up to 94 % enantiomeric excess (*ee*), and 20 : 1 diastereomeric ratio (d.r.)]. Mechanistically, the organic photocatalyst **17** was capable of producing reactive benzyl radicals from trifluoroborate precursors (**1**), while the Ni^0^ catalyst was involved in the activation of the starting materials **14** through an oxidative addition, prior to intercepting the so‐formed radicals yielding a Ni^III^ species. Lastly, the final keto‐acids **15** were released from the complex via reductive elimination. It is worth noting that the described mechanism is in analogy with the previously proposed Ni/metallaphotoredox catalytic pathway, and this corroborates the functionality of organic chromophores as a replacement for noble metal‐based systems.

**Figure 9:**
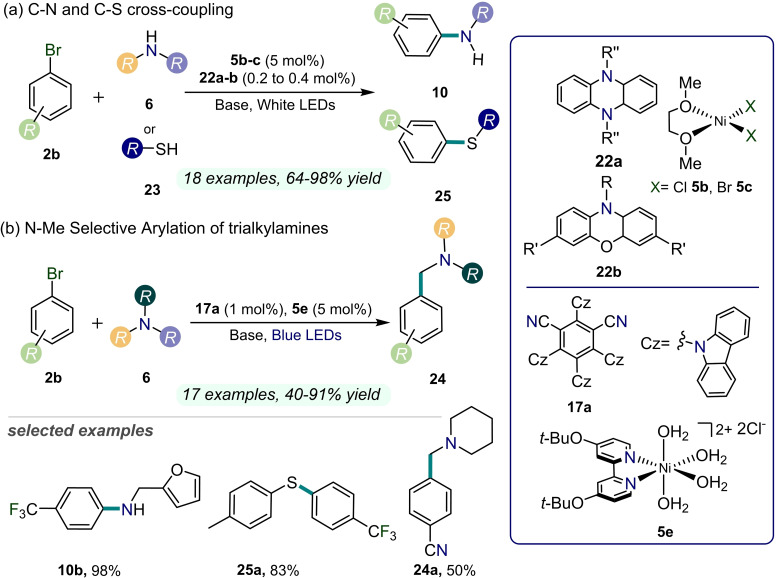
Dual Ni/organophotoredox‐catalyzed C−heteroatom cross‐couplings between aryl halides **2** and amines **6** or thiols **23**, described in Refs. [64,65].

In the same year, Miyake and co‐workers described C−N and C−S bond‐forming reactions with aryl bromides **2b** by using a dual Ni/organophotocatalytic system (Figure 9).^[64]^ They exploited the ability of highly reducing dyes, namely dihydrophenazines and phenoxazines **22 a**,**b** [−1.69 and −1.80 V vs. saturated calomel electrode (SCE), respectively], to trigger hetero‐coupling reactivities in combination with a Ni^II^ source **5 b**,**c** under visible light irradiation. Thereby, the corresponding aniline and sulfide derivatives **10** and **25** were efficiently obtained under mild conditions by shining white light (up to 98 % yield). Interestingly, these transformations could be carried out on multigram scale or even under natural sunlight. From a photophysical point of view, the organic dyes **22 a**, **b** employed in this work turned out to possess comparable redox properties of the common polypyridyl Ru and Ir complexes employed for the described coupling reactions.^[33]^ Remarkably, in this example one of the lowest photocatalyst loading has been exploited within the realm of Ni/organophotoredox catalysis, although not yet close to the extremely small amounts of the best‐performing Ir‐based systems.^[33]^

In 2021, Shen and Rovis published a late‐stage functionalization protocol for the *N*‐methyl arylation of trialkylamines enabled by dual Ni/organophotoredox catalysis (Figure 9).^[65]^ The authors envisaged the use of 4‐CzIPN (**17**) as organic dye together with a Ni^II^ metal complex **5 e** to achieve the selective production of pharmaceutically relevant derivatives under blue light irradiation. A variety of aryl bromides **2 b** were effectively coupled with different amines **6** to generate the corresponding benzylamines **24** under mild reaction conditions (up to 91 % yield). A simplified mechanistic interpretation of the studied reactivity involved the formation of α‐amino radicals from the starting amines **6** by means of the light‐excited chromophore **17**. Then, the so‐obtained open‐shell species were intercepted by the Ni^II^ complex, leading to the formation of highly reactive Ni^III^ intermediates that finally underwent reductive elimination to give rise to the desired products **24**. The outstanding selectivity of the illustrated mechanistic route facilitates the synthesis of fundamental building blocks in pharmaceutics, along with opening the way to smarter drug discovery.

**Figure 10:**
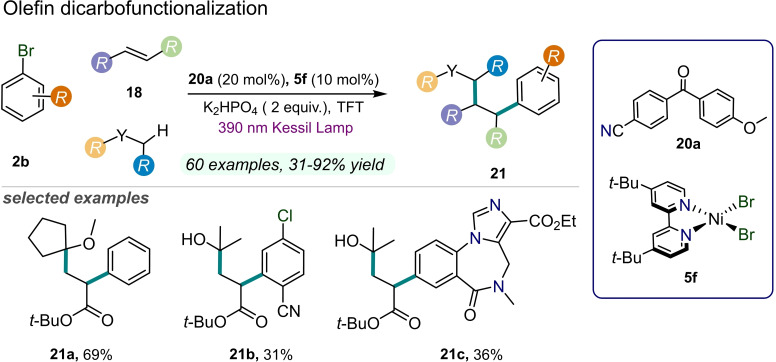
Dual Ni/organophotoredox‐catalyzed olefin dicarbofunctionalization, described in Ref. [66]. TFT=α,α,α‐trifluorotoluene.

Recently, a dual photochemical C−H activation methodology for the dicarbofunctionalization of olefins **18** was achieved through Ni catalysis (Figure 10).^[66]^ A combination of a diaryl ketone as organophotoredox catalyst **20 a** and a Ni^II^ complex **5 f** allowed the activation of native C−H bonds of commodity reagents that become capable of interacting with aryl bromides **2 b** and alkenes **18** in a three‐component reaction.

As a result, the corresponding dicarbofunctionalized products **21** were obtained in a one‐pot reaction under strong UV light irradiation (390 nm, up to 92 % yield). Remarkably, natural‐based substrates can be converted by using this methodology. In spite of the intricate mechanism of this transformation, the authors were able to demonstrate how the process is initiated by the photoexcitation of the diaryl ketone **20 a** that triggered the formation of a carbon‐centered radical from an activated C−H bonds of the corresponding precursors. Then, the alkyl radical undergoes regioselective Giese addition to the olefin **18** and, subsequently, is captured by a Ni^0^ species. The resulting Ni^I^ complex undergoes oxidative addition to Ni^III^ with the aryl bromide **2 b** followed by reductive elimination generating the desired dicarbofunctionalized product **21**. These mechanistic considerations were corroborated by dispersion‐corrected DFT calculations, additionally highlighting the strong dependence of the studied reactivity on the relative concentration of nickel catalyst **5 f** to alkene **18**.

An alternative approach to typical dual Ni/organophotoredox methodologies consists in a direct photochemical Ni‐catalyzed process without the aid of any external photocatalyst. This concept, recently proposed by Melchiorre and co‐workers, exploits the photochemical properties of a specific class of substrates, namely 4‐alkyl‐1,4‐dihydropyridines, which can produce carbon‐centered radicals upon direct irradiation. The so‐generated species can therefore participate in the Ni catalytic cycle along with the other reaction partners, thus leading to the corresponding functionalized products.[^67^, ^68^, ^69^] Remarkably, when a chiral Ni complex is employed, the coupling reactivity occurs in an enantioselective fashion with good stereocontrol.^[69]^ These latter examples are based on a more classical photochemical procedure to access highly reactive intermediates. Nevertheless, their stricter substrate‐dependence and a poorer atom economy should be taken into account.

As highlighted in the previous examples, such dual Ni/organophotoredox systems represent effective and noble metal‐free routes to produce relevant organic molecules. However, the limitations of molecular organic photocatalysts both in terms of recovery and possible photobleaching may limit their industrial applicability.

## 4 Dual Ni/Heterogeneous Photoredox Catalysis

Heterogeneous semiconductor materials have been less investigated for photoredox catalytic reactions compared to homogeneous counterparts, but in the last years they have shown promising results in synthetic chemistry.^[70]^

In 2017, Weix and co‐workers reported nanosized CdSe quantum dots as robust and efficient partners with a nickel catalyst for the amination of aryl halides **2**.^[71^ Similarly, a reusable CdS semiconductor has been exploited for C−N and C−O cross couplings by Xiao and co‐workers.^[72^ Currently, heterogeneous materials with lower toxicity have become the main target, inspiring massive research. In the just outlined panorama, the class of carbon nitride (CN) photocatalysts is complying with most of the guidelines of sustainable catalyst development thanks to its metal‐free and non‐toxic nature. Recently, the attention of the scientific community for these semiconductors has grown enormously for visible‐light photocatalytic application in organic synthesis.[^45^, ^46^] Graphitic carbon nitride (*g*‐CN) is a metal‐free polymer and 2D material with a bandgap of approximately 2.7 eV. It is an effective and well‐studied semiconductor for a wide range of reactions in organic photocatalysis, as CN is able to generate surface redox centers as electron–hole pairs upon visible‐light photoexcitation, allowing two aligned redox processes to occur at the same time.^[44]^ It can be easily obtained in multigram quantities by thermal condensation of cheap molecular precursors such as melamine, urea, or cyanamide.

CN material is made up by defect‐rich repeating N‐bridged poly(tri‐s‐triazine) units arranged into graphite‐like π‐conjugated planar layers with a theoretically C/N ratio of 3 : 4. Graphitic carbon nitride is a robust photocatalyst, recyclable and reusable multiple times. Interestingly, the redox properties of CNs can be tuned to make them suitable for a broad light‐driven transformations by modulating, for example, the C/N ratio, the crystallinity, the polymerization degree, the surface area, or the presence of doping agents (e. g., B, S, O, organic additives).[^73^, ^74^]

**Figure 11:**
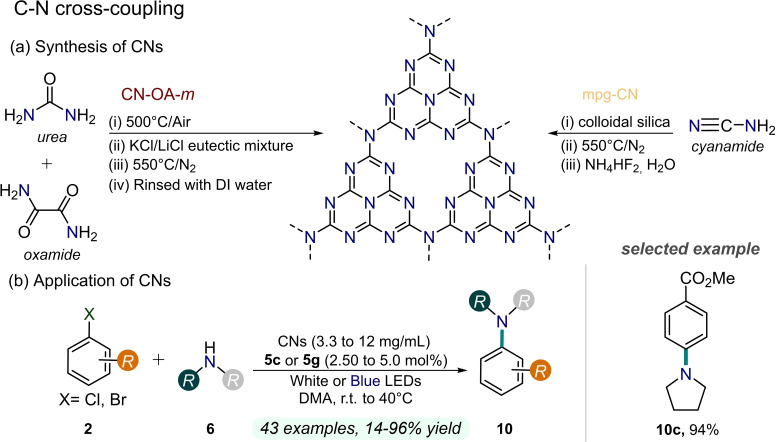
Dual Ni/CNs photocatalysis for C−N bond‐forming reactions, described in Refs. [75, 76].

Although CN materials have been investigated in organic photocatalysis over the last decade, König and co‐workers reported the first relevant example of dual Ni/heterogeneous photoredox catalysis in 2019.^[75]^ In this regard, the authors exploited inexpensive mesoporous graphitic carbon nitride (*mpg*‐CN) as heterogeneous photocatalyst combined with a Ni catalyst for the functionalization of arenes **2** under blue light irradiation (Figure 11). The *mpg*‐CN was prepared from cyanamide as precursor by thermal treatment at 550 °C in the presence of mesoporous silica matrices, subsequently removed with ammonium hydrogen difluoride in water. In addition to various investigated reactivities, the authors reported CN/Ni dual catalytic C−N couplings between aryl halides **2** and amines **6** to obtain aniline products **10** in excellent yields (up to 92 % yield) with the presence of NiBr_2_ ⋅ glyme catalyst **5 c** under blue light irradiation (455 nm) in dimethylacetamide (DMA) as solvent. The developed protocol required the use of a high photocatalyst loading (12 mg mL^−1^) to ensure the described reactivity. The synthetic scope involved electron‐poor aryl bromides **2 b** and even aryl chlorides **2 c**. As anticipated, the key advantage in utilizing a heterogeneous photoredox catalyst relies on the easy and effective recyclability of this material over multiple sequential reactions. Indeed, in this work mesoporous carbon nitride was smoothly recovered by simple centrifugation and reused several times with conserved activity.

In 2020, Pieber and co‐workers employed a different carbon nitride‐based material prepared by the co‐condensation of urea and oxamide followed by post‐calcination in presence of KCl and LiCl, namely CN‐*OA‐m*, to study highly selective nickel/photoredox‐catalyzed aryl aminations (Figure 11).^[76]^ The authors observed that the deposition of nickel‐black deactivated the heterogeneous PC. This issue could be overcome by decreasing the rate of reductive elimination, increasing the oxidative addition rate, or stabilizing low‐valent nickel intermediates. Notably, this work examined the influence of the incident light wavelength to hamper the nickel‐black formation. To this aim, the use of green light (520 nm), rather than blue (450 nm) or white light turned out to be more effective, thus improving the CN‐OA‐*m* recyclability, although the required reaction time was consequently drastically extended (up to 168 h). The proposed strategies enable the C−N cross‐coupling of electron‐rich, electron‐neutral, and electron‐poor aryl halides **2** with primary and cyclic secondary amines **6** in high selectivity (up to 96 % yield), using NiBr_2_ ⋅ 3H_2_O **5 g** as nickel source and relatively low CN‐OA‐*m* loading (3.3 mg mL^−1^). Remarkable results were achieved even by employing electron‐poor aryl chlorides **2 c**.

These C−N coupling reactions have been successfully scaled‐up by means of an oscillatory plug‐flow reactor, capable of handling solid/liquid mixtures in continuous flow, achieving the preparation of pharmaceutically relevant precursors with high productivity in multigram scale.^[77]^ Moreover, C−heteroatom and C−C bond formation reactions using a nickel catalyst on dye‐sensitized titanium dioxide are an effective strategy, where the dye‐sensitization allows for to the use of full‐visible light spectrum, and can overcome excited state lifetime limitations of PCs.^[78]^

**Figure 12:**
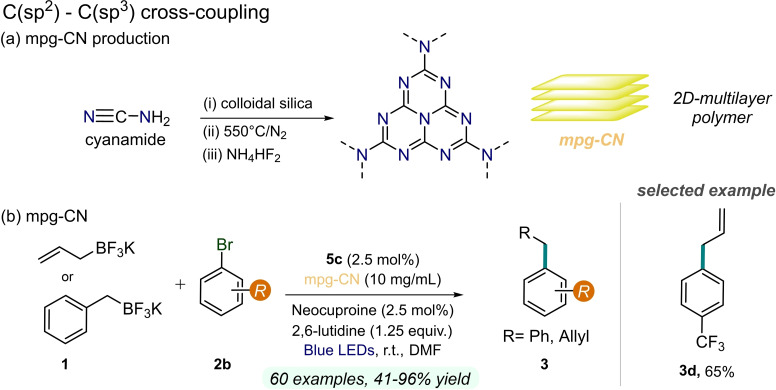
Dual Ni/CNs photocatalysis for C−C coupling reaction, described in Ref. [79].

In the same year, König and co‐workers described an organoboron cross‐coupling reaction under blue light irradiation using *mpg*‐CN as heterogeneous photocatalyst and an available and stable Ni source **5 c** (Figure 12). The strategy is compatible with various electron‐donating, electron‐withdrawing, and (hetero)arenes **2**. Moreover, not only benzyl trifluoroborates have been investigated, but also allyl trifluoroborates **1**, although in both cases a fairly high PC loading has been employed (10 mg mL^−1^). This expansion of the reaction scope allows the installation of functionally important allyl groups onto arenes.^[79]^ In addition, the expected recyclability of *mpg*‐CN was verified over different consecutive cycles with perfectly preserved activity and reaction rates.

**Figure 13:**
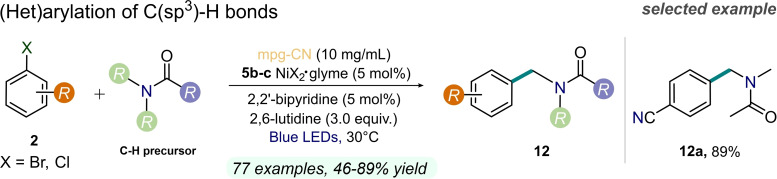
Dual Ni/CN photocatalysis for C−H functionalization reactions, described in Ref. [80].

The use of *mpg*‐CN as photoredox catalyst in tandem with nickel catalysis for the direct functionalization of C(sp^3^)−H bonds under blue light irradiation was investigated by the same research group (Figure 13).^[80]^ The system required a relatively high photocatalyst loading (10 mg mL^−1^) and fairly long reaction times (up to 48 h). Catalytically generated halogen radicals on the *mpg*‐CN semiconductor surface can act as hydrogen atom transfer (HAT) agents for the formation of carbon‐centered radicals that would allow the arylation reactions. Mechanistic studies with a pre‐formed *ortho*‐tolyl‐Ni^II^ catalyst provided insights towards an energy‐transfer process (EnT) occurring between light‐excited *mpg*‐CN and the Ni species. This photochemical step generates electronically excited Ni^II^ complex as a reactive intermediate to drive the studied chemistry. The authors exploited the described mechanism to activate C−H bonds adjacent to the nitrogen atom of amide groups, since *N*‐benzyl amides **12** represent a common moiety in pharmaceuticals. In this way, a wide variety of aryl halides **2** with electron‐withdrawing groups and moderately electron‐donating substituents were effectively converted into the corresponding amides **12** (up to 89 % yield) by using a relatively high loading of both *mpg*‐CN and additives. Moreover, this method has been applied to the late‐stage functionalization of bioactive aryl components, including pharmaceuticals and agrochemicals (up to 91 % yield).

In 2021, *mpg*‐CN was employed with Ni^II^ salts to divergently synthesize 1,4‐dicarbonyl compounds or substitute alkenes though Mizoroki–Heck cross‐couplings under blue LED irradiation.^[81]^ The protocol demonstrated high functional group tolerance, affording the corresponding coupling derivatives in good yield and at gram scale (up to 87 % yield). The employed *mpg*‐CN turned out to be fully recoverable and recyclable several times. Remarkably, this protocol required a very low amount of Ni catalyst (down to 1.25 mol %). Recently, Liu et al. proposed a sulfur‐ and a sulfur‐boron‐doped carbon nitride with vacancies for different organic transformations under blue LED irradiation.^[82]^ A broad array of C−N, C−O, and C−S cross‐coupling reactions were performed with Ni catalysis to evaluate the effect on the reactivity of the structural features of the employed CN materials. The authors performed a comparison between previously reported CN photocatalysts (e. g., *g*‐CN, *mpg*‐CN, etc.) and their doped materials in a series of model transformations. In the selected reaction conditions, the S‐ and B‐doped CNs turned out to be more effective than the related heterogeneous photocatalysts.

**Figure 14:**
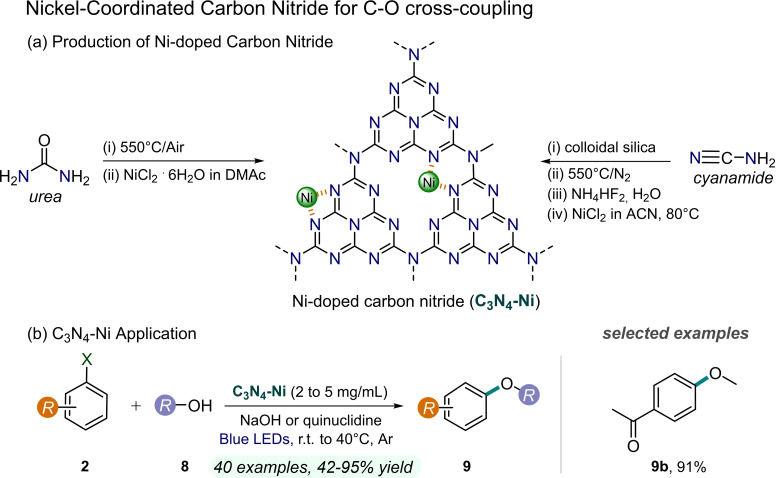
Ni‐doped carbon nitride photocatalyst for C−O coupling reactions, described in Refs. [86–88].

As mentioned in the introduction, an attractive prospect is to apply the principle of dual catalysis to SAC, where the Ni complex is heterogenized by installing the Ni atom directly onto the PC surface. However, the choice of the support for the SAC is critical as it must be able to stabilize the isolated metal and prevent its aggregation (favored by the high surface and cohesive energy of the single metal) without compromising its activity by interfering too heavily with the metal electronic configuration.^[83]^ g‐CN seems to be one of the ideal structures for stabilizing single metal sites by means of its extended N‐rich interstitial structure, without jeopardizing the metal catalytic ability.[^84^, ^85^] The great expected advantage of SAC is surely the direct electronic communication between the Ni and the PC, avoiding possible problems of diffusion‐control kinetics of the Ni complex to the PC surface. First inspiring examples on this approach have been very recently reported. Zhao et al. developed in 2020 a preassembled CN−Ni platform for C−O cross‐coupling of aryl halides **2** and alcohols **8** under strong white light irradiation (Figure 14).^[86]^ The composite catalyst consists of a mononuclear Ni anchored on carbon nitride, prepared by direct coordination of Ni^II^ to CN nitrogen, with imidazole as auxiliary ligand. It is worth noting that the nickel content determined by X‐ray photoelectron spectroscopy (XPS) is around 1 wt %, greater than the value obtained by inductively coupled plasma mass spectrometry (ICP‐MS), hence suggesting that most Ni atoms are located on the CN surface. A further insight into the Ni arrangement was provided by computational studies, which highlighted how the Ni atoms reside into the two‐dimensional CN plane. Additionally, these theoretical investigations revealed that the imidazole ligand serves to provide an enhanced accessibility of the metal to the reaction partners. Visible‐light driven etherification of aryl halides **2** with large excess of alcohols or, alternatively, hydroxylation with water in presence of quinuclidine exhibited high turnover number (TON), selectivity, and yield (up to 97 % yield). A remarkably low amount of Ni is therefore involved in the reactivity, since only 2 mg mL^−1^ loading of CN−Ni‐**1** is required. Moreover, the heterogeneous nature of the photocatalyst allows its easy recyclability, although longer reaction times were required to preserve its catalytic activity. This can be attributed to a detectable decrease in the Ni content of CN−Ni‐**1** over the different cycles. Nonetheless, the authors excluded any possible dominant contribution from homogeneous Ni catalysis to the overall reactivity through a series of control experiments.

In the following year, Reisner and co‐workers developed a Ni‐deposited mesoporous graphitic carbon nitride (CN−Ni‐**2**) to promote with a synergic effect C−O bond formation reactions between alcohols **8** and a variety of aryl halides **2** under blue LED irradiation (447 nm, Figure 14).^[87]^ CN−Ni‐**2** catalyst was prepared by heating to 80 °C a mixture of NiCl_2_ and *mpg*‐CN in acetonitrile with triethylamine under microwave treatment. Deposited nickel (4 wt % by ICP analysis) was found to be uniformly present as Ni^2+^ active site and did not affect the morphology, composition, and optical properties of *mpg*‐CN. This integrated heterogeneous photocatalyst represented a simple and sustainable approach which allowed a broad and effective C−O coupling substrate scope (up to 92 % yield), although the alcohol reagents **8** have to be employed as solvent. Mechanistic and computational investigations suggested that, upon light excitation of CN−Ni‐**2**, photogenerated holes are quenched by alcohols, while photoexcited electrons are transferred from *mpg*‐CN to Ni sites through internal photoinduced electron transfer (PET), yielding Ni^I^ species; subsequently, a Ni^I^/Ni^III^ cycle occurs, consisting of oxidative addition, ligand exchange, and final reductive elimination to complete the catalytic cycle and to obtain the ether product **9**. The recyclability of CN−Ni‐**2** was assessed over three cycles, whereas a significant drop in the activity was observed since the fourth run.

Very recently, the same research group presented another use of CN−Ni‐**2** for the selective photochemical preparation of primary anilines by using sodium azide as source of nitrogen.^[88]^ These latest examples demonstrate the ever‐growing interest of the scientific community towards the development of novel applications of “all‐in‐one” heterogeneous photocatalysts. As in the previous study, the deposited nickel has a fairly high loading of 5.9 wt % by ICP analysis.

## 5 Critical Aspects in Designing Ni/Heterogeneous Semiconductor Dual Catalysis

The critical aspects in designing dual photocatalytic systems based on heterogeneous semiconductors are manifold. The required interaction between the homogeneous Ni complex and the solution‐dispersed semiconductor (SC) implies a diffusion of the Ni towards the SC's surface. At a first glance, it is expected that such a diffusion must be as facile as possible to allow fast catalytic kinetics; however, it is important to consider that the “dark” catalytic cycle is ideally performed by the homogenous Ni complex. If a too fast diffusion is accompanied by high rates of formation of photo‐excited electrons, the build‐up of adsorbed Ni ions on the SC surface may lead to progressive deposition of Ni^0^ nanoparticles, which is one of the typical deactivation pathways in this type of photocatalysis.^[76]^ Hence, the rates of diffusion and of photogenerated charge formation must be opportunely tuned in order to orchestrate the diffusion→adsorption→electron transfer→desorption sequence and secure the most efficient progress of the reaction.

At this point, it becomes obvious that the choice of the solvent is of paramount importance not only to control diffusion rates of the Ni complex, but also to generate the most appropriate complex species. Too strongly coordinating solvents may inhibit the adsorption of the Ni complex, which is hypothesized to take place by means of coordinating moieties of the SC (e. g., functional groups, unsaturated lattice atoms, etc). On the other hand, a solvent molecule that is a too labile ligand may be completely replaced by other coordinating molecules, such as the reactants (amines, thiols, alcohols) and compromise the formation of the Ni complex with the right orbital configuration for participating in the electron transfer process. Computational analysis is a powerful tool that can provide help to define the most suitable coordination environment of the Ni center, and so allow to find the most appropriate reaction conditions.

Fundamental to the development of Ni/SC‐based catalytic schemes is the understanding of the true mechanism of catalysis. The electron transfers from (or to, if the SC acts as electron acceptor) the SC to the Ni may occur either after adsorption of the Ni on the surface or in solution via mediating molecular radicals.^[40]^ In the latter case, the tuning of the Ni diffusion needs to be readjusted. Another possibility is that the reaction occurs directly on the SC‐bound Ni, so that the catalysis is fully heterogeneous.^[43]^ In this case, the design of the catalytic systems must take into account that the Ni precursor and the complex species to be generated (i. e., the reaction conditions) should be tailored to attain a bound state of the Ni center. Not only the materials, but the end purpose also has to be carefully evaluated.

Synthetic organic chemistry often addresses biologically relevant molecules as the final target, which can have applications in the synthesis of medical products. To this end, attention must be placed to the fact the Ni is a common allergenic metal, and even in small doses may give severe side‐effects when introduced into the body.^[89]^ Large organic molecules synthesized with this approach may have high affinity to bind Ni and retain the metal even after work up. It would be desirable to use accurate characterization of the isolated product to check for complete Ni removal.

## 6 Summary and Outlook

The multi‐faceted application potential of the dual catalysis makes this strategy highly flexible and versatile, deemed to be one of the dominant synthetic approaches for organic synthesis in the near future. The use of nickel as the active metal, and of light as the energy source to control the evolution of the Ni oxidation states during the catalytic cycle relieves the constrains of the poor cost‐effectiveness of noble metal catalysts. Moreover, the photocatalytic nature ensures a higher level of sustainability of the overall process, in compliance with modern requirements for the chemical industry.

Several possibilities arise depending on the choice of the photoredox species: the use of metal complex as the photoredox catalyst (PC) represents the traditional and virtually the most effective methodology. Indeed, dual Ni/metallaphotoredox catalysis operates in extremely low loadings, down to parts per million (ppm) level. Nevertheless, this approach is being replaced by more economic and sustainable alternatives.

The line of development has featured the rise of organic dyes as an option to ensure high activity at a lower cost, ideally envisioning the substitution of rare metal‐based photocatalysts with chromophores possessing very similar photoredox properties.

Finally, heterogeneous photocatalysts, based on non‐metal semiconductors, are perhaps the currently most investigated pathway, with graphitic carbon nitride (*g*‐CN) being undoubtedly the protagonist. While the advantageous features of *g*‐CN are well established, future development will have to focus on the design of experiments to shed more light on the mechanism of action. In particular, the inhomogeneous nature of *g*‐CN implies that the exact sub‐structure that interacts with the Ni complex is difficult to define, so that a rational structural tailoring is not straightforward. Understanding the exact functionality to fine‐tune electron transfer dynamics and coordination to the Ni center is of critical importance for securing the definitive advent of heterogeneous metal‐free PC. Advanced spectroscopy, in situ and operando, combined with theoretical calculations seems to be the inevitable plan of action.

The possibility of creating an “only‐heterogeneous” dual catalyst by inserting the Ni atoms directly on the surface of the photocatalyst (as to make single‐atom catalysts) is maybe the frontier in dual catalysis organic chemistry. Apart from the obvious benefit of recyclability, the intimate relationship between the Ni and the PC could have important effects on reaction kinetics and selectivity. Moreover, the fact that the catalyst is a single material can make the characterization and mechanistic understanding easier as opposed to a dual hetero‐/homogeneous type of catalyst, where the transient Ni species and their exact diffusion/interaction to/with the photocatalyst are more complicated to unravel.

Lastly, the underexplored photochemical Ni‐catalyzed enantioselective transformations could be the subject of the main focuses of synthetic chemists in the near future.[^90^, ^91^] As a matter of fact, the possibility of creating optically active compounds in a stereocontrolled fashion by means of sustainable, efficient, and fully recyclable dual Ni/photocatalytic system would represent the final goal of this prominent field of research. In particular, the combination of enantiopure chiral ligands that are capable of coordinating Ni atoms and generating effective chiral metal complexes, and suitable photo‐active carbon nitride‐based photocatalytic systems could lead to the production of valuable enantioenriched organic compounds. This strategy has been largely unexplored to date. We thus expect that those imminent investigations on such topics will contribute to determine the future directions in organic synthesis for both academia and industry.

## Conflict of interest

The authors declare no conflict of interest.

## Biographical Information

*Giacomo Filippini obtained his Master's degree in Industrial Chemistry from the University of Bologna (Italy). In 2013 he joined the group of Prof. Paolo Melchiorre at ICIQ in Tarragona (Spain), where he undertook his doctoral studies. In 2017, he started a postdoctoral appointment in the group of Prof. Maurizio Prato at University of Trieste (Italy), where he is currently Assistant Professor, investigating the use of carbon‐based nanomaterials to design novel organic transformations*.

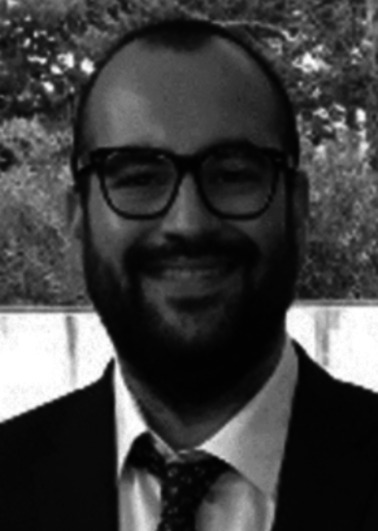

## Biographical Information

*Maurizio Prato is Professor of Organic Chemistry at the University of Trieste and Ikerbasque Research Professor at CIC biomaGUNE, Spain. He was the recipient of two ERC Advanced Research Grant, European Research Council, in 2008 and 2020 and became a Member of the National Academy of Sciences (Accademia Nazionale dei Lincei) in 2010. His research focuses on the synthesis of innovative carbon‐based functional materials for applications in materials science, nanomedicine, and catalysis*.

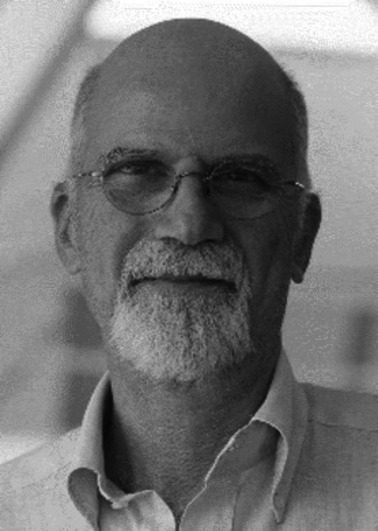

## Biographical Information

*Paolo Fornasiero is professor of inorganic chemistry at the University of Trieste. His research focuses on the application of inorganic chemistry in nanoscience for the preparation of nanomaterials for catalysis and energy related applications. He is co‐author of more than 300 ISI publications, 19 book chapters, and 4 patents. He received the 2005 Nasini Gold Metal, the 2013 Chiusoli Gold Medal, the 2016 Heinz Heinemann Award, the 2017 Kramer Award, the 2018 M. T. Messori Roncaglia Award and the 2022 L. Malatesta Medal*.